# Elevated Plasma Von Willebrand Factor and Propeptide Levels in Malawian Children with Malaria

**DOI:** 10.1371/journal.pone.0025626

**Published:** 2011-11-17

**Authors:** Happy T. Phiri, Daniel J. Bridges, Simon J. Glover, Jan A. van Mourik, Bas de Laat, Bridon M'baya, Terrie E. Taylor, Karl B. Seydel, Malcolm E. Molyneux, E. Brian Faragher, Alister G. Craig, James E. G. Bunn

**Affiliations:** 1 Malawi-Liverpool-Wellcome Trust Clinical Research Programme, College of Medicine, Blantyre, Malawi; 2 Blantyre Malaria Project, College of Medicine, University of Malawi, Blantyre, Malawi; 3 Departments of Plasma Proteins and Blood Coagulation, Sanquin, Amsterdam, The Netherlands; 4 Malawi Blood Transfusion Services, Blantyre, Malawi; 5 Department of Internal Medicine, College of Osteopathic Medicine, Michigan State University, East Lansing, Michigan, United States of America; 6 Liverpool School of Tropical Medicine, Liverpool, United Kingdom; 7 College of Medicine, Blantyre, Malawi; Institut Pasteur, France

## Abstract

**Background:**

In spite of the significant mortality associated with *Plasmodium falciparum* infection, the mechanisms underlying severe disease remain poorly understood. We have previously shown evidence of endothelial activation in Ghanaian children with malaria, indicated by elevated plasma levels of both von Willebrand factor (VWF) and its propeptide. In the current prospective study of children in Malawi with retinopathy confirmed cerebral malaria, we compared these markers with uncomplicated malaria, non malarial febrile illness and controls.

**Methods and Findings:**

Children with cerebral malaria, mild malaria and controls without malaria were recruited into the study. All comatose patients were examined by direct and indirect ophthalmoscopy. Plasma VWF and propeptide levels were measured by ELISA. Median VWF and propeptide levels were significantly higher in patients with uncomplicated malaria than in children with non-malarial febrile illness of comparable severity, in whom levels were higher than in non-febrile controls. Median concentrations of both markers were higher in cerebral malaria than in uncomplicated malaria, and were similar in patients with and without retinopathy. Levels of both VWF and propeptide fell significantly 48 hours after commencing therapy and were normal one month later.

**Conclusions:**

In children with malaria plasma VWF and propeptide levels are markedly elevated in both cerebral and mild paediatric malaria, with levels matching disease severity, and these normalize upon recovery. High levels of both markers also occur in retinopathy-negative ‘cerebral malaria’ cases, many of whom are thought to be suffering from diseases other than malaria, indicating that further studies of these markers will be required to determine their sensitivity and specificity.

## Introduction

Falciparum malaria is one of the commonest potentially fatal infections in sub-Saharan Africa. Most deaths from malaria occur in young children living in areas of intense *Plasmodium falciparum* transmission. African children bear 90% of the brunt of mortality from falciparum malaria, with high case fatality rates in children with cerebral malaria even with good treatment [1]. Falciparum malaria is a major cause of morbidity and mortality in Malawi particularly in children under the age of five. More than 40% of in-hospital deaths in Malawian children are attributed to malaria, and cerebral malaria is a common manifestation of severe and complicated malaria [2]. Children in Blantyre district, where transmission is stable and intense, suffer on average 2.8 symptomatic malaria episodes per year while adults, though still susceptible to infection, are less likely to have symptomatic disease [3].

*In vivo* von Willebrand Factor (VWF) biosynthesis is limited to endothelial cells and megakaryocytes. VWF is produced and released by vascular endothelial cells, and is frequently used as an indicator of endothelial cell activation in vascular disorders such as thrombotic thrombocytopenic purpura (TTP) and sepsis [4]–[6]. Published work has demonstrated that VWF plays a critical role in primary haemostasis by mediating platelet adhesion to sites of vascular injury [7], [8]. Before newly synthesized VWF leaves the endothelial cell, it undergoes endoproteolytic cleavage of its propeptide. The processed VWF and propeptide are either released constitutively or, following activation of the endothelium, released through a regulated pathway. Upon stimulation, mature VWF and propeptide are released in equimolar amounts and, once in the blood, mature VWF and its propeptide become completely dissociated and have different lifespans [9], [10]. VWF propeptide is important for intracellular trafficking and processing of VWF [4], [11], [12] and is cleared from the circulation at a much faster rate than mature VWF, with a half-life of approximately two hours. VWF, together with its propeptide, is stored within Weibel Palade bodies [13] and exists as ultra large VWF multimers with a half-life of 10–12 hours. The multimeric composition of VWF is a critical determinant of its functional activity. Both mature VWF and propeptide concentrations significantly increase upon acute perturbation of the endothelium [14]. Since VWF propeptide has a more rapid turnover than VWF, measurement of the concentrations of both molecules in the peripheral blood allows discrimination between chronic and acute phases of endothelial cell activation *in vivo*.

In a prospective study of patients from Ghana with different malaria presentations, we showed that patients with severe *P. falciparum* infection had significantly increased plasma VWF and propeptide levels, consistent with acute endothelial cell activation [15]. Work has shown that elevated plasma VWF and propeptide levels develop soon after the onset of a *P falciparum* parasitaemia [16], indicating that acute endothelial cell activation constitutes an early feature of this infection. Indeed our recent findings show that severe *P. falciparum* malaria is associated with circulating ultra large VWF multimers [17], consistent with acute endothelial cell activaton. We have also demonstrated that rapid activation of endothelial cells enables *P. falciparum* adhesion to platelet-decorated VWF strings [18], a novel mechanism by which *P. falciparum*-infected erythrocytes (IE) might sequester in vascular beds which do not express appropriate host endothelial receptors. These findings together suggest the possibility that the secretion of VWF by activated endothelial cells may be a mechanism that enhances the severity of *P falciparum* malaria.

Severe malaria is commonly misdiagnosed in Africa, because a patient with severe disease of any cause may have a parasitaemia that is coincidental and not responsible for the illness [19]. Malarial retinopathy is a set of retinal signs [20], some of which are unique to malaria and, on the basis of post-mortem studies [21], when identified in a comatose patient, greatly strengthen the confidence with which the syndrome can be attributed to malaria.

In the current prospective study we investigated plasma VWF and propeptide levels in paediatric malaria. We measured mature VWF and propeptide concentrations in plasma samples collected from children with *P. falciparum* parasitaemia who had severe disease or uncomplicated febrile illness and controls without parasitaemia. We used malarial retinopathy to differentiate patients with definite cerebral malaria from those with encephalopathy accompanied by parasitaemia.

## Materials and Methods

### 1. Patient selection

The study was carried out at Queen Elizabeth Central Hospital (QECH) in Blantyre, Malawi. Children with *P falciparum* parasitaemia presenting with uncomplicated febrile illness or with encephalopathy were recruited for the study. Controls were febrile children without parasitaemia and well children undergoing routine surgery. Children (mean age = 33 months) were recruited for both the control and index groups after their parents or guardians had given written informed consent to their participation in the study. Clinical details were recorded for all cases at the time of recruitment, and all subsequent analyses were carried out blind to these details. All patients with malaria received standard supportive and anti-malarial treatment.

#### Cerebral malaria cases

Patients (n = 100) were recruited when admitted with an initial clinical diagnosis of cerebral malaria (CM), defined as a Blantyre Coma Score (BCS) of 2 or less [21], with peripheral *P. falciparum* parasitaemia and no other identifiable cause of coma [22]. CM children were examined for retinopathy [20]. In nine CM children with retinopathy an additional sample was collected at 48 hours, and in seven a further sample was collected 30 days following admission at a follow up visit as part of a separate study.

#### Mild malaria cases

Patients with mild malaria (MM) were children attending the same hospital's Paediatric Accident and Emergency Unit (PAEU). These children were febrile and parasitaemic, had no complications suggestive of severe malaria [23], and were well enough to be managed at home (n = 59).

#### Febrile controls without malaria

Children (6–60 months) with a non-malaria febrile illness (NMFI) were recruited from among attenders at the same PAEU. This group comprised children not requiring admission, who had a documented febrile illness (>37.5°C) with no malaria parasites on a single thick blood film, a negative rapid diagnostic test for malarial antigens (Paracheck, Orchid Biomedical Systems, Goa, India), and no history of having taken any antimalarial medication in the previous two weeks (n = 32).

#### Healthy controls

These were well children with no febrile illness (NFI) admitted for elective surgery (n = 20). A Paracheck malaria rapid antigen diagnostic test was performed on all controls and only those negative were included in the analysis (n = 17).

#### Adult controls

To develop a Malawian standard for VWF and propeptide, and a normal range for a Malawian (adult) population, we recruited 50 healthy Malawian adult blood donors through the Malawi Blood Transfusion Services (MBTS). These donors were negative for both human immunodeficiency virus (HIV) by serology and for malaria by thick blood film microscopy and rapid diagnostic test. Citrated plasma samples were pooled together to provide a local standard.

### 2. Clinical measurements

Clinical details were taken using a research proforma at the time of admission, and for children with CM through timed data capture points during ward admission. Retinal examination in CM was performed by one ophthalmologist with extensive previous experience of observing malarial retinopathy. Mydriatic eyedrops were used in all cases, and the retina was examined by both direct and indirect ophthalmoscopy.

### 3. Laboratory measurements

For the measurements of plasma VWF and VWF propeptide in hospital and emergency room patients and for the MBTS blood donors, venous blood samples were collected in citrated tubes (1∶9 vol/vol), and immediately placed at 4°C until spun. The 30 days post-admission samples were collected in lithium heparin. Samples were centrifuged and plasma was aliquoted and stored at −80°C until analysed batchwise.

VWF and VWF propeptide concentrations were measured by enzyme-linked immunosorbent assay (ELISA) as previously described [23]. Pooled plasma from 40 adult Caucasian donors containing 49 nM of VWF and 5.5 nM of VWF propeptide was used as a standard on each plate. Plasma lactate was measured by Lactate-Pro analyser. EDTA blood was used for preparing a Fields stained thick blood film which was examined microscopically to determine parasitaemia. Beckman Coulter Hmx Hematology analyzer was used to measure haemoglobin and platelet count. Children with CM were tested for HIV using two rapid diagnostic test kits, rapid Uni-Gold HIV test (Trinity Biotech PLC, Ireland) and Determine HIV rapid test (Abbott Laboratories, USA).

### 4. Ethical approval

This study was approved by the ethics committees of the College of Medicine, University of Malawi and Liverpool School of Tropical Medicine.

### 5. Statistical analysis

Data were analyzed using the GraphPad Prism v4.03 and SPSS (version 18) statistical packages. As all of the continuous variables had very positively skewed distributions, all analyses were conducted using distribution-free statistical tests; results are summarised using medians (ranges) unless otherwise stated. Plasma concentrations of VWF and propeptide were compared across the study sub-groups using the Kruskal-Wallis test with Bonferroni correction for post-hoc multiple pairwise testing. The Friedman test was used to compare median plasma concentrations of VWF and propeptide in children during and after treatment. Comparisons of groups based on malaria retinopathy, illness outcome, HIV status and gender were made using the Mann-Whitney U-test. Correlations between variables were estimated using the Spearman correlation coefficient. Statistical significance was set at the conventional 5% level for all analyses.

## Results

### Patients' characteristics

A total of 100 children with CM were recruited, and 99 underwent ophthalmology examination, with 79 (79.8%) having retinopathy. Of these, 13 children died, and nine were discharged with clinically significant neurological deficits. The remaining children responded to antimalarial drug therapy and made a full recovery. The clinical and laboratory findings in different subsets of children with and without *falciparum* malaria are summarized in Table 1.

**Table 1:** Clinical and laboratory characteristics by diagnosis of children with and without *Plasmodium falciparum* malaria – medians (ranges).

	CM Cerebral Malaria		MM Mild malaria	NMFI Non-malaria febrile illness controls	NFI Non-febrile illness controls
	retinopathy positive	retinopathy negative			
Sample size	79	20	59	32	17
Sex^1^ female	32 (40.5%)	11 (55.0%)	26 (44.1%)	12 (38.7%)	8 (47.1%)
male	47 (59.5%)	9 (45.0%)	33 (55.9%)	19 (61.3%)	9 (52.9%)
Age (months)^2^	34.5 (8.0–86.0)	36.5 (17.0–164.0)	24.0 (3.0–60.0)	23.5 (6.0–60.0)	23.0 (6.0–48.0)
Lactate (nM)	6.9 (1.1–18.9)	5.1 (1.2–21.4)	n.t.	n.t.	n.t.
Platelets (10^4^/µl)^3^	5.2 (0.5–26.6)	12.5 (0.6–54.5)	13.8 (2.9–31.6)	34.1 (27.4–58.0)	n.t.
Haemoglobin (g/dl)^4^	6.1 (2.5–10.0)	9.2 (4.3–12.9)	7.9 (3.6–12.8)	10.3 (7.0–12.5)	n.t.
Parasitaemia (10^3^/µl)^5^	51.5 (0.1–1149.4)	58.8 (0.2–725.9)	n.t.	n.t.	n.t.
VWF (nM)	191.1 (40.5–484.3)	171.9 (110.1–427.2)	117.2 (64.4–355.3)	72.3 (20.4–142.7)	37.1 (9.3–62.1)
VWFpp (nM)	28.4 (4.8–107.5)	33.2 (9.0–80.0)	18.3 (5.1–53.2)	9.3 (2.3–22.3)	3.4 (1.0–30.6)

n.t.: not tested.

1 not recorded for 1 child in NMFI group.

2 not recorded for 1 child in CM retinopathy positive group.

3 not recorded for 5 children in CM retinopathy positive group, 1 child in CM retinopathy negative group, 10 children in MM group and 22 children in NMFI group.

4 not recorded for 10 children in MM group and 22 children in NMFI group.

5 not recorded for 2 children in CM retinopathy positive group and 1 child in CM retinopathy negative group.

Normal range for lactate: 0.7–2.1 nM.

### Plasma VWF and propeptide concentrations in Malawian children

**Figure 1:**
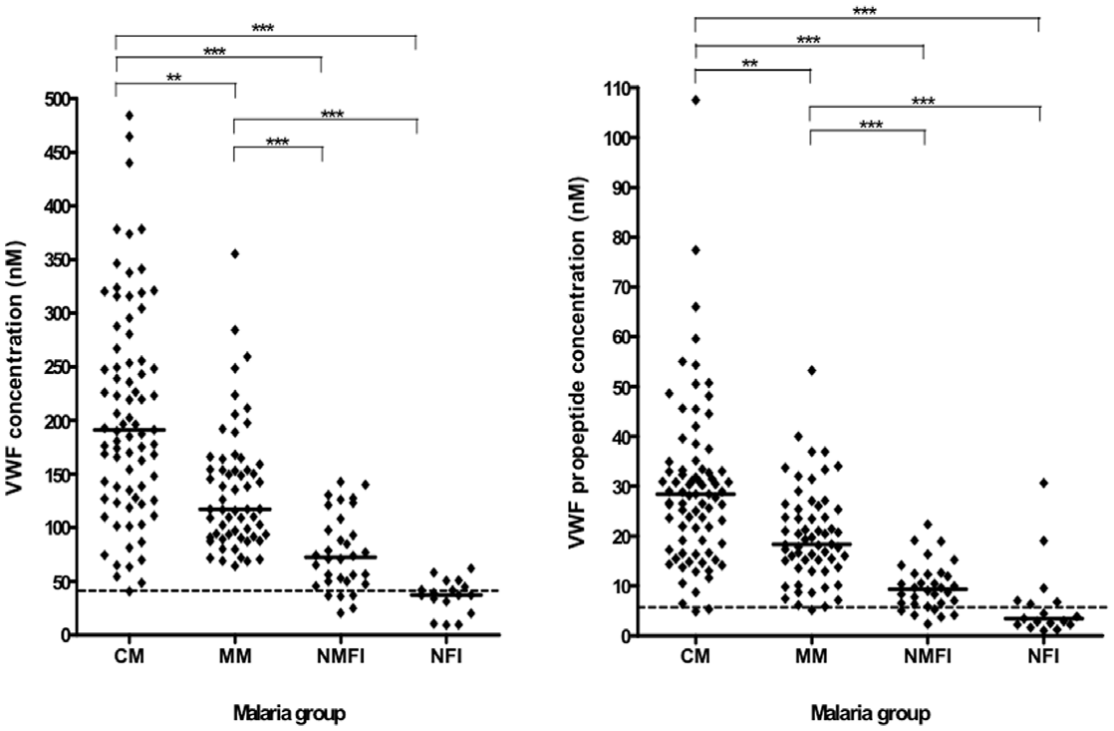
Plasma concentration of von Willebrand factor (VWF) and VWF propeptide in various subsets of children with and without *Plasmodium falciparum* malaria. Graphs showing median and scatter of plasma VWF and VWF propeptide levels in children with cerebral malaria (CM) and mild malaria (MM), and in non-malaria febrile illness (NMFI) and non-febrile illness (NFI) controls as measured by ELISA (Kruskal-Wallis, *** P<0.001; **P<0.01 after Bonferroni correction for post-hoc multiple pairwise testing). Dotted lines indicate median levels in local healthy Malawian adults.

The median plasma concentrations of VWF and propeptide of the NFI controls were 37.1 (9.3–62.1) nM and 3.4 (1.0–30.6) nM, respectively (Figure 1). These values are within the normal range of healthy Caucasians [23], [24] and local healthy Malawians (median: 42.9 (20.4–110.6) nM and 5.1 (1.8–15.0) nM for VWF and propeptide, respectively). In NMFI children, median VWF and propeptide levels were 72.3 (20.4–142.7) nM and 9.3 (2.3–22.3) nM, respectively, values that were significantly different from normal values for healthy Caucasian and Malawian subjects (p<0.001 for both variables).

Median plasma concentrations of both VWF and propeptide at admission were significantly higher in children with both cerebral and mild malaria when compared with the non-malarial control groups (p<0.001 both variables) - and were also significantly higher in children with cerebral malaria compared to those with mild malaria (p<0.001 for both variables, Figure 1).

### Plasma VWF and propeptide concentrations during and after treatment

**Figure 2:**
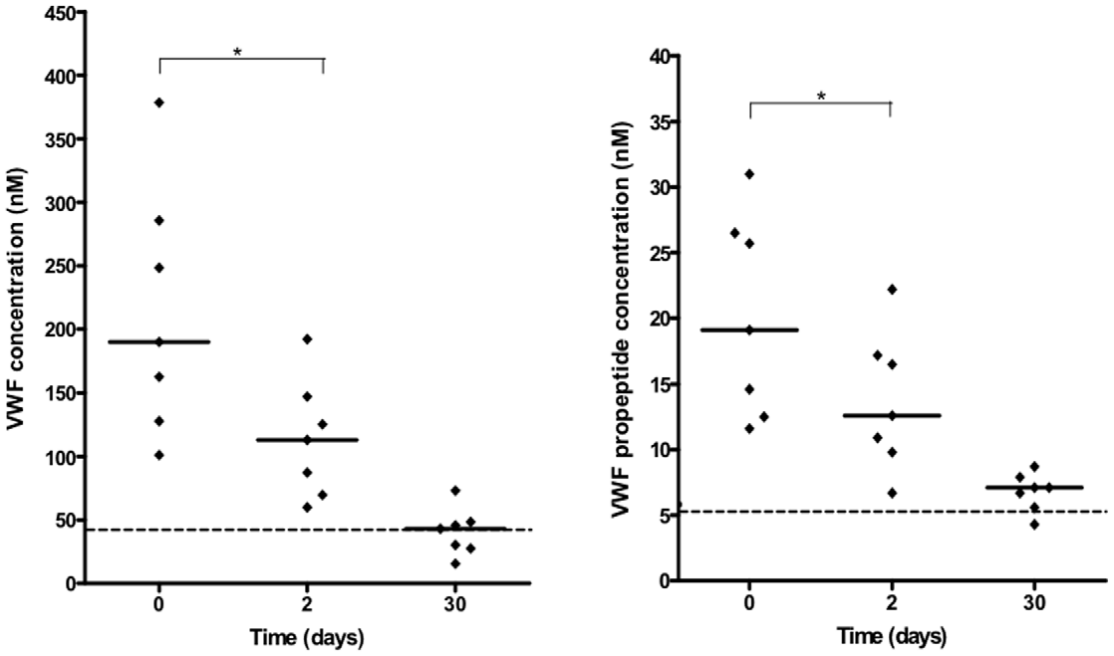
Plasma concentration of VWF and propeptide in *Plasmodium falciparum* cerebral malaria patients during and after treatment. Plasma levels of VWF and VWF propeptide were measured at admission, 2 days and 30 days post-treatment in a cohort of retinopathy positive children with cerebral malaria (Friedman Test, *P<0.05). Dotted lines indicate median levels in local healthy Malawian adults.

A subset of cerebral malaria children (n = 7) was studied prospectively to relate plasma VWF and propeptide levels at the time of admission to plasma levels 48 hours and 30 days after the start of treatment. At 48 hours, median plasma VWF and propeptide levels had dropped significantly from 189.9 (101.0–378.4) to 112.9 (59.8–192.0) nM and 19.1 (11.6–31.0) to 12.6 (6.7–22.2) nM, respectively (p = 0.016 for both variables, Figure 2).

After recovery (30 days after receiving therapy), median VWF and propeptide levels in this subset of children were significantly lower than at admission (p = 0.016 for both variables) or at 48 hours (p = 0.016 for VWF, p = 0.031 for propeptide) (Figure 2), with median plasma levels at 30 days similar to normal levels in controls.

### VWF and propeptide levels in children diagnosed with cerebral malaria, with and without retinopathy

**Figure 3:**
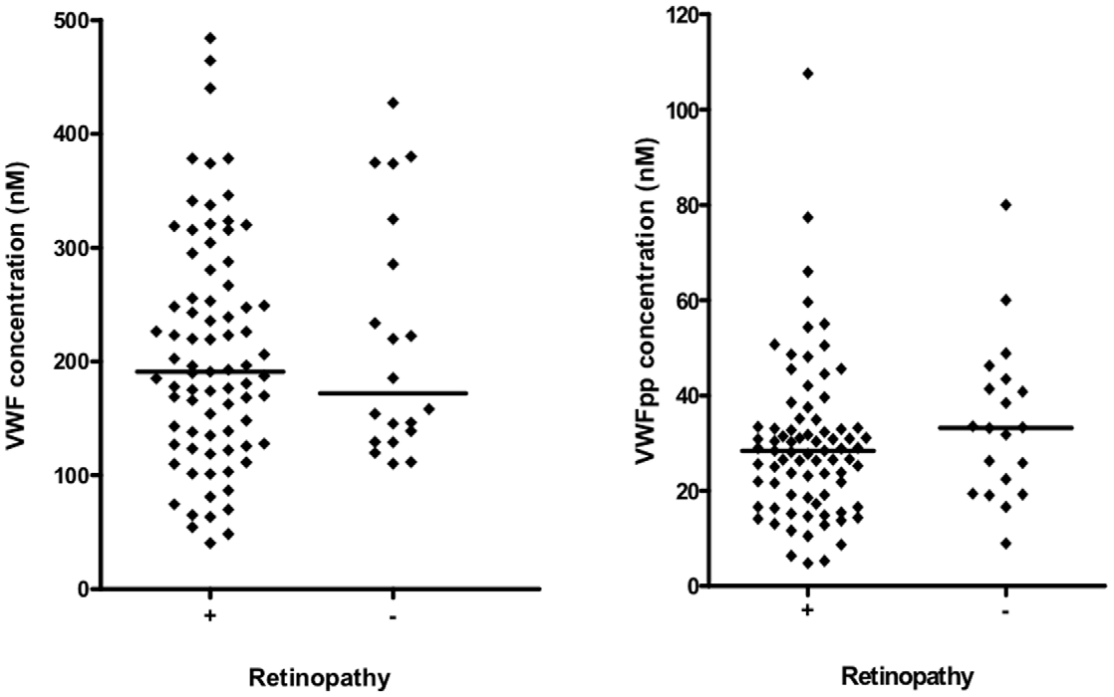
Plasma concentration of VWF and propeptide in severe *Plasmodium falciparum* malaria patients with and without retinopathy. Graphs showing median and scatter of plasma VWF and VWF propeptide levels in cerebral malaria children with (+) and without (−) retinopathy as measured by ELISA (Mann Whitney U test, p = 0.832 and p = 0.143, respectively).

VWF and propeptide plasma concentrations were compared between cerebral malaria children with retinopathy and those without retinopathy. Median concentrations and ranges of the two proteins were similar in these two groups (p = 0.832 and p = 0.143, respectively, Figure 3).

### VWF and propeptide levels in retinopathy-positive CM according to clinical outcome

**Figure 4:**
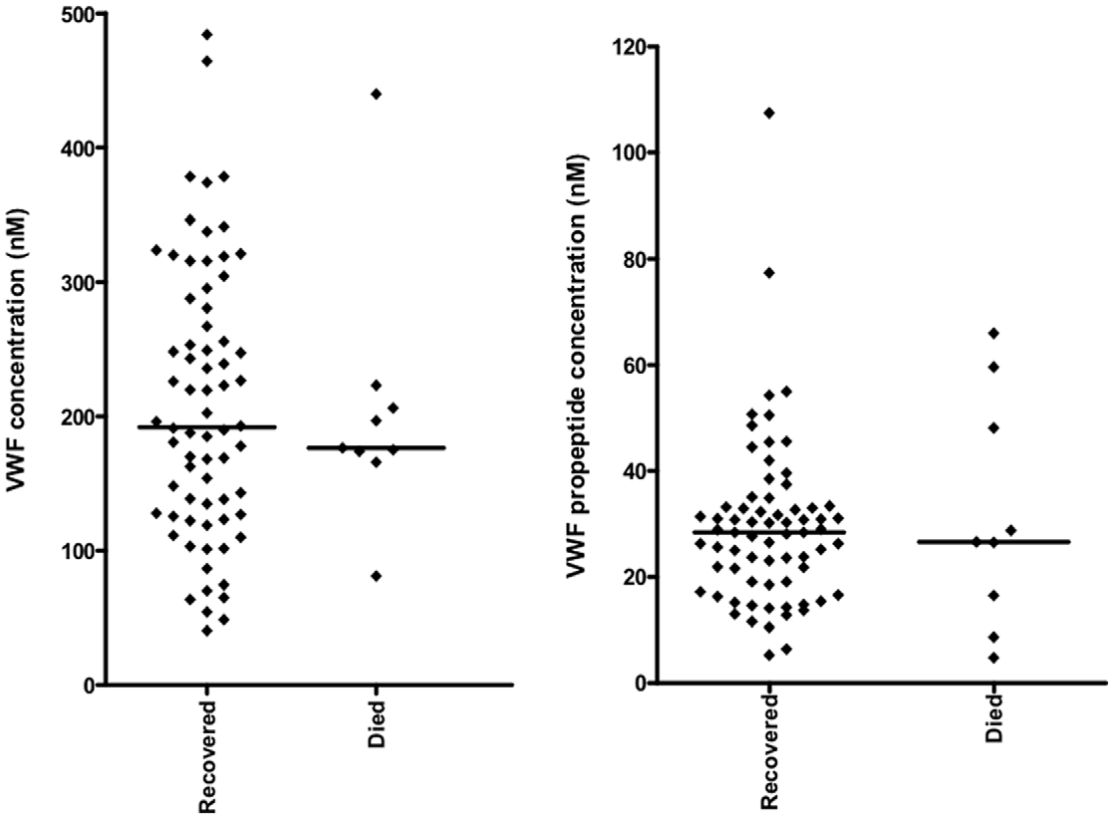
Plasma concentration of VWF and propeptide in *Plasmodium falciparum* cerebral malaria patients with retinopathy. Graphs showing median and scatter of plasma VWF and VWF propeptide levels in retinopathy positive children with cerebral malaria who died (Died) or recovered (Recovered) as measured by ELISA (Mann Whitney U test, p = 0.873, p = 1.000, respectively).

To determine whether VWF and propeptide are prognostic markers for death in confidently-diagnosed paediatric cerebral malaria, we measured plasma protein levels in retinopathy positive children who recovered and those who died. Median plasma VWF levels did not differ significantly between these two groups: survived: 192.0 (40.5–484.3), died 176.5 (81.1–440.1); p = 0.873. Similarly, median propeptide levels did not differ significantly: survived 28.4 (5.3–107.5), died 26.6 (4.8–66.0); p = 1.000 (Figure 4).

### Other clinical measurements

The median circulating platelet count differed significantly between the NMFI group and the cerebral and mild malaria groups (p<0.001 for both comparisons, Table 1). Platelet counts were significantly lower in children with cerebral malaria than in those with mild malaria (p<0.001). Platelet count did not correlate with either VWF or VWF propeptide in children with retinopathy positive cerebral malaria (rho = −0.165, p = 0.160 and rho = −0.074; P = 0.533, respectively). Children with retinopathy positive cerebral malaria had lower median (range) platelet counts 52,000 (5000–266,000) than those without retinopathy 125,000 (6000–545,000) (Mann Whitney U Test p = 0.056).

Of the 69 children with cerebral malaria and positive retinopathy for whom HIV status was known, 59 were sero-negative, and ten had positive HIV serology. Median concentrations in each for VWF were 196.6 (54.3–484.3) and 153.5 (48.5–247.4) nM respectively, and for propeptide were 30.2 (4.8–107.5) and 25.4 (6.4–44.5) nM (p = 0.059 and p = 0.137 respectively).

Neither VWF nor propeptide concentration correlated with plasma lactate levels (rho = −0.198; p = 0.080 and rho = −0.020; p = 0.862 respectively). There was no significant difference in median VWF or propeptide concentration between children with lactic acidosis (plasma lactate >5 nM) (VWF: 224.5 (63.4–378.4); propeptide: 26.4 (4.8–50.7) and those with plasma lactate <5 nM (VWF: 185.0 (40.5–484.3); propeptide: 28.4 (5.3–107.5) (p = 0.340 and p = 0.504 respectively). Median VWF and propeptide levels did not differ between male children (n = 47; VWF: 202.5 (40.5–484.3); propeptide: 26.6 (4.8–107.5)) and female children (n = 32; VWF: 182.0 (54.3–378.4); propeptide: 29.9 (11.6–77.4)) children (p = 0.418 and p = 0.168 respectively).

## Discussion

We studied the patterns of increase in the plasma concentrations of VWF and propeptide in Malawian children with mild and severe malarial disease in order to assess the relationship of these biomarkers with disease severity. Our findings show that VWF and propeptide levels are markedly elevated in patients with retinopathy positive cerebral malaria but less so in mild malaria (Figure 1). In some patients VWF and propeptide levels were even higher than the levels published in known cases of fulminant vascular disorders such as TTP and sepsis [14]. Our observations corroborate those of Hollestelle *et al*., [15] that circulating concentrations of both VWF and its propeptide are specifically and markedly raised in patients with *P. falciparum* infection, and that VWF and propeptide levels are markers of endothelial activation in falciparum malaria, at higher levels than seen in other infections, suggesting a specific perturbation in malaria.

The recovery of cerebral malaria patients was associated with a significant decline in both VWF and propeptide levels towards normal values in healthy Malawian controls (Figure 2). Since levels of VWF returned to normal population values following infection, inherent levels of VWF do not appear to be raised in these children. One surprising observation different from that seen in Ghana [15] was that neither VWF nor propeptide levels correlated with blood lactate levels in our cohort, with no significant difference in VWF and propeptide levels between children with hyperlactataemia (lactate >5 nM) and those with blood lactate concentrations <5 nM. Plasma lactate has been reported to be of pathophysiological and prognostic significance in both children and adults with cerebral malaria [25], [26].

A novel aspect of this study is the assessment of plasma VWF and propeptide levels in cerebral malaria cases confirmed on the basis of malarial retinopathy. The presence of malarial retinopathy in histopathological studies of fatal paediatric cerebral malaria has been reported to have a positive predictive value of 95% and a specificity of 90% for the detection of histopathologically confirmed cerebral malaria in parasitaemic patients [27]. Therefore in research studies it is the best technique yet available to confirm that a patient has cerebral malaria, and exclude from the cohort those who clinically appear to have cerebral malaria but might actually have another underlying cause for their coma.

The observation that plasma VWF and propeptide levels were not significantly different between children who died and those who recovered (Figure 4) suggests that these proteins are not prognostic markers of death in cerebral malaria. Recent observations have shown that that, in addition to other biomarkers, VWF propeptide is a good marker of cerebral malaria in fatal paediatric malaria and that both VWF and propeptide are good at differentiating between cerebral and mild malaria [28], [29]. The surprising result from our study comes from the comparison of VWF and propeptide levels in cases defined clinically as cerebral malaria but divided into retinopathy positive and negative. Our assumption is that the retinopathy negative cases represent very sick children in a coma but where the parasitaemia is incidental, rather than the underlying cause of pathology. The similar levels of VWF and propeptide in both categories suggest that VWF levels are not able to discriminate between cerebral malaria and coma due to other causes in the presence of malaria parasites. One possible explanation for this would be that increased VWF levels are seen generally on infection by the malaria parasite, as demonstrated in mild malaria cases but a second component not linked directly to parasitaemia but perhaps linked to general pro-inflammatory processes or endothelial dysfunction may be responsible for the further increase seen in cerebral malaria. Thus the very high levels of VWF and propeptide seen here might be markers of severe pathology (e.g. coma) with malaria infection rather than specifically cerebral malaria. Further studies comparing parasitaemic and non-parasitaemic severe disease cases will be required to discriminate between these components.

Platelets were significantly lower in patients with cerebral malaria than in those with mild malaria. Platelet changes have long been known in malaria, thrombocytopenia being a usual feature of plasmodial infections. Several independent lines of evidence suggest that platelets play a role in the pathogenesis of cerebral malaria: (1) postmortem studies in African children with cerebral malaria have revealed that in some cases platelets co-localize with sequestered IE at brain microvascular sites, suggesting that platelets can play a role in the pathogenesis of cerebral malaria; (2) *in vitro* studies have demonstrated platelet-mediated IE adhesion to TNF-activated brain endothelium by acting as a bridge between the endothelium and IE [30], a mechanism that allows CD36-mediated binding in microvessels where CD36 expression is known to be low or irregular [31]; (3) platelets have been implicated in IE clumping associated with pathogenesis of malaria [32]. Also our recent studies in Malawi have established a novel mechanism by which IE adhere via platelet decorated ultra large VWF strings on activated endothelium. Large VWF multimers in plasma bind activated platelets with ∼100 fold higher affinities compared to monomers [33], [34]. As well as enabling IE to sequester in vascular beds which do not express appropriate IE receptors, this novel mechanism also provides a link between the observed high levels of VWF in malaria and platelet-mediated IE adhesion to brain endothelium. This mechanism may explain the low platelet count observed in children with malaria, and in particular retinopathy positive cerebral malaria patients in our cohort due to extensive platelet sequestration in the vasculature. Furthermore the malaria parasite may be able to modulate the production of VWF through release of an orthologue of Translationally Controlled Tumor Protein (PfTCTP), promoting histamine release from basophils (*in vitro*), and present in early asymptomatic infection in humans (*in vivo*) [34].

In summary, we have confirmed that plasma VWF and propeptide levels are markedly elevated in both cerebral and mild paediatric malaria, discriminate between these two conditions and that in cerebral malaria these normalize upon recovery. It is not possible to say whether VWF and VWF propeptide are more markedly elevated specifically in retinopathy positive cerebral malaria than other central nervous system (CNS) severe illnesses in the presence of malaria infection. Further work on the specificity and sensitivity of VWF and VWF propeptide is therefore required to ascertain their role as prognostic biomarkers for malaria disease severity.
